# Nucleosomes Correlate with *In Vivo* Progression Pattern of *De Novo* Methylation of *p16* CpG Islands in Human Gastric Carcinogenesis

**DOI:** 10.1371/journal.pone.0035928

**Published:** 2012-04-25

**Authors:** Zhe-Ming Lu, Jing Zhou, Xiuhong Wang, Zhenpo Guan, Hua Bai, Zhao-Jun Liu, Na Su, Kaifeng Pan, Jiafu Ji, Dajun Deng

**Affiliations:** 1 Key Laboratory of Carcinogenesis and Translational Research, Ministry of Education, Division of Cancer Etiology, Peking University Cancer Hospital and Institute, Beijing, China; 2 Department of Epidemiology, Peking University Cancer Hospital and Institute, Beijing, China; 3 Department of Surgery, Peking University Cancer Hospital and Institute, Beijing, China; Vanderbilt University Medical Center, United States of America

## Abstract

**Background:**

The exact relationship between nucleosome positioning and methylation of CpG islands in human pathogenesis is unknown.

**Methodology/Principal Findings:**

In the present study, we characterized the nucleosome position within the *p16* CpG island and established a seeding methylation-specific PCR (sMSP) assay based on bisulfite modification to enrich the *p16* alleles containing methylated-CpG at the methylation “seeding" sites within its intron-1 in gastric carcinogenesis. The sMSP-positive rate in primary gastric carcinoma (GC) samples (36/40) was significantly higher than that observed in gastritis (19/45) or normal samples (7/13) (*P*<0.01). Extensive clone sequencing of these sMSP products showed that the density of methylated-CpGs in *p16* CpG islands increased gradually along with the severity of pathological changes in gastric tissues. In gastritis lesions the methylation was frequently observed in the region corresponding to the exon-1 coding-nucleosome and the 5′UTR-nucleosome; the methylation was further extended to the region corresponding to the promoter-nucleosome in GC samples. Only few methylated-CpG sites were randomly detected within *p16* CpG islands in normal tissues. The significantly inversed relationship between the *p16* exon-1 methylation and its transcription was observed in GC samples. An exact *p16* promoter-specific 83 bp-MSP assay confirms the result of sMSP (33/55 vs. 1/6, *P*<0.01). In addition, *p16* methylation in chronic gastritis lesions significantly correlated with *H. pylori* infection; however, such correlation was not observed in GC specimens.

**Conclusions/Significance:**

It was determined that *de novo* methylation was initiated in the coding region of *p16* exon-1 in gastritis, then progressed to its 5′UTR, and ultimately to the proximal promoter in GCs. Nucleosomes may function as the basic extension/progression unit of *de novo* methylation of *p16* CpG islands *in vivo*.

## Introduction

Methylation of CpG islands around transcription start sites (TSS) represses gene expression epigenetically and plays crucial roles in cell differentiation, development, and pathogenesis. It has previously been reported that nucleosome positioning may influence DNA methylation patterns throughout the genome, and DNA methyltransferases preferentially target nucleosome-bound DNA in the *Arabidopsis thaliana* and human embryonic stem cells [1]. However, the mechanistic details describing the role of nucleosome positioning in relation to DNA methylation are still unknown. Attempts to characterize exact aberrant methylation and its extension patterns within CpG islands in human tissue samples, especially in the early stage of carcinogenesis, have not been successful for variety of reasons. Problems arise from the fact that only a very small fraction of cells undergo *de novo* methylation (extension) in cellular heterozygous tissues making detection difficult, and the sensitivity limits of current approaches are unable to determine the methylation and demethylation patterns dynamically.

Tumor suppressor gene *p16* (CDKN2A) is a cell cycle regulator involved in inhibiting the G1→S phase transition [2]. Aberrant methylation of CpG islands is the main mechanism for *p16* inactivation in multiple human cancers [3]–[5]. *p16* Methylation is an early event in carcinogenesis and has been shown to significantly increase the risk of malignant transformation of epithelial dysplasia in the stomach, oral cavity, and other organs in followup cohort studies [6]–[9]. In fact, it is undergone to develop the aberrant *p16* methylation as a prognosis predictor for precancerous lesions [10], [11].

Although *p16* methylation is one of the well-studied epigenetic events [12]–[16], most of our knowledge on this event originated from studies using *in vitro* cell culture systems, in which full methylation of *p16* CpG islands has been established. However, full methylation is not often detectable in tissue samples, especially in the early stages of carcinogenesis. Although methylation-specific PCR (MSP) or MethyLight assays targeted to the *p16* exon-1 region are the most widely used assays [11], [17], [18], the natural methylation patterns of each CpG site within this CpG island in human tissues with/without pathological changes have not been comprehensively studied at a single molecule level.

It has been reported that *p16* methylation is very stable in cultured cancer cell lines based on its efficient recovery after the removal of a DNA methylation inhibitor treatment [19]. In contrast, most *p16* methylation in gastritis lesions is unstable and *H. pylori*-dependent as indicated by the 150 bp-MSP assay [20], [21]. The mechanisms contributing to the difference in stability of *p16* methylation between gastritis and cancer cells is unknown. Characterization of the natural methylation pattern of *p16* CpG islands in human tissue samples of various pathological states may elucidate the mechanisms accounting for the diverse stability of *p16* methylation and could potentially be used to develop a tumor-specific methylation biomarker assay. In the present study, we explored the relationship between the natural methylation signatures of *p16* CpG islands and nucleosome positioning in human gastric carcinogenesis.

## Materials and Methods

### Tissue samples and *H. pylori* status

Surgical GC samples from 68 patients (49 males and 19 females, 35–81 years old), chronic gastritis biopsies from 45 patients (33 males and 12 females, 18–65 years old) and gastric mucosa biopsies from 13 healthy male subjects (18–47 years old) were obtained from the Peking University Cancer Hospital/Institute. All these tissues were histologically verified. The Ethnics Committee of the Peking University Cancer Hospital/Institute approved the study. The written, informed consent was obtained from all subjects. The *H. pylori* status was determined using a PCR assay to amplify *H. pylori*-specific *23S rDNA* as described [22].

### Cell culture

Human cancer cell line MGC803 was cultured in RPMI 1640 medium with 10% FBS at 37°C and 5% CO_2_. AGS (ATCC# CRL-1739) was cultured in F12 medium with 10% FBS. MGC803 and AGS cell lines were kindly provided by Prof. Yang Ke at the Cancer Institute [23]. We reported the *p16* methylation and expression data of these two cell lines previously [13].

### DNA preparation, bisulfite treatment and clone sequencing

Genomic DNA was extracted from tissue samples with phenol/chloroform. The unmethylated cytosine of the genomic DNA was converted to uracil through treatment with 5 mol/L sodium bisulfite for 16 hrs at 50°C [24]. Bisulfite genomic sequencing was used to analyze the methylation patterns of individual DNA molecules. PCR products were amplified from the bisulfite-treated DNA templates with primer sets complementary to the deaminated DNA as described below. The amplicons were cloned into the pCR-blunt vector (Invitrogen, Carlsbad, CA), transformed into *E. coli*, and sequenced using an ABI 3730 Analyzer. At least 20 clones were then randomly selected and sequenced for each sample. To demonstrate the initiation and extension of *de novo* methylation of *p16* CpG islands, the methylation status of each clone was analyzed as a categorical variable positive: methylation level ≥5%, that is when the methylated CpG sites in a analyzed fragment (including 48 tested CpG sites) in the *p16* CpG island is ≥3, it is defined as initially methylated molecules. Otherwise, we designated it as methylation-negative.

### Relative enrichment (chromatin accessibility) measured by quantitative PCR

**Figure 1:**
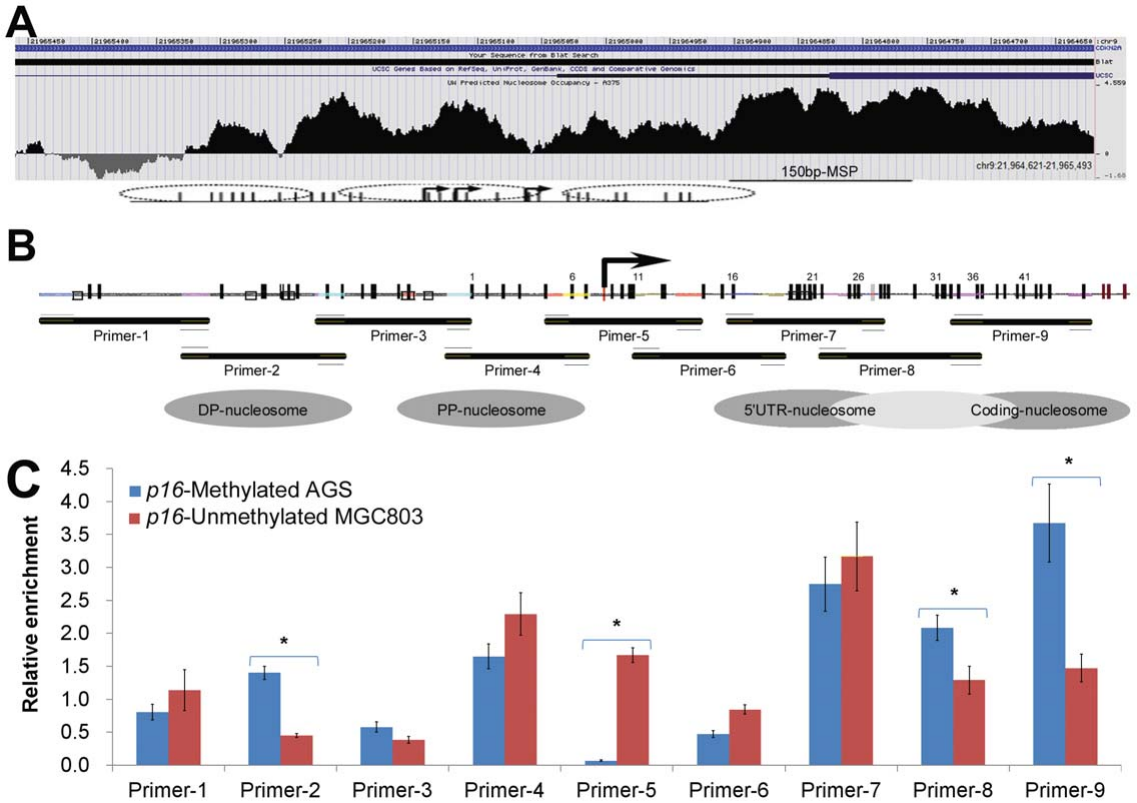
Chromatin accessibility determined by quantitative PCR across the *p16* promoter. (**A**) Genomic organization of the *p16* promoter and exon-1 region; Predicted nucleosome occupancy in A375 cells are depicted as black peaks and the fragment without nucleosome signal are depicted as inverse gray peaks [29]. The previously reported nucleosome position (three ovals) and location of the 150 bp-MSP amplicon (black line) are also illustrated [15], [17]. (**B**) Amplicons of the chromatin accessibility measured through a set of quantitative PCR assays were used to quantify the mono-nucleosomal DNA partially digested using *MNase* (Horizontal lines). The detected nucleosomes are marked using gray ovals. Positions of these CpG sites in the *p16* genomic sequence corresponding to the fragment displayed in panel A are indicated by thin vertical lines, and the transcription start site (TSS) is represented as a bent arrow. (**C**) The relative enrichment of various fragments within *p16* CpG island in *p16*-methylated AGS and *p16*-active MGC803 cells with and without digestion using 0.1 U *MNase* for 5 min. Relative enrichment was calculated and normalized according to the relative copy number (2^−ΔCt^; ΔCt = Ct^Uncut^−Ct^Cut^) for each primer set [24], [26]. The average value and standard deviation of four quantitative PCR reactions are displayed. We reported the *p16* methylation and expression data of these two cells previously [13].

To determine the relationship between methylation and the nucleosome occupancy in *p16* CpG islands, a set of quantitative PCR assays were performed in a similar manner to previously published studies with minor modifications [25]–[28]. The mono-nucleosomal DNA from *p16*-methylated AGS and *p16*-unmethylated MGC803 cell lines was isolated using the EZ nucleosomal DNA Prep Kit (Zymo Research, Orange, CA) according the manufacturer's instructions. Briefly, the procedure includes cell nuclei isolation, intact nuclei micrococcal nuclease (*MNase*) digestion, and mono-nucleosomal DNA purification. DNA recovered from the *MNase* ‘Cut’ and ‘Uncut’ control samples was used in quantitative PCR assays to measure the relative enrichment of targeted regions using a series of primer pairs (Fig. 1b and 1c; Table S1). For each primer set, the forward and reverse primers were designed to cover within 147 bp of the template sequence, which theoretically constitutes the core DNA region of a nucleosome. Each PCR was performed in duplicate. The following quantitative PCR conditions were used: 95°C for 15 min, and 45 cycles of 95°C for 10 s, 62°C for 20 s, and 72°C for 20 s, followed by 72°C for 10 min. All PCR reactions were performed using the power SYBR Green PCR Master Mix (P/N 4367659, Applied. Biosystems, Foster City, CA) on an ABI-7500 Real-Time PCR system (Applied Biosystems). Relative enrichment was calculated according to the relative copy number (2^−ΔCt^; ΔCt = Ct^Uncut^−Ct^Cut^) for each primer set [24], [26]. Thus, *MNase*-hypersensitive fragments in nucleosome-free regions (with high chromatin accessibility) such as linker DNA were depleted in the *MNase*-treated samples and gave lower relative enrichment than those with the *MNase*-hyposensitive ones (with low accessibility) such as coral nucleosomal fragments.

### PCR amplifications

CpG-free forward and reverse primers (5′-gtagg tgggg aggag tttag tt-3′ and 5′-ctacc taatt ccaat tcccc taca-3′) were used to amplify both the methylated and unmethylated *p16* CpG islands (588 bp). The same region was also amplified by sMSP (forward primer, 5′-gtagg tgggg aggag tttag tt-3′; reverse primers, 5′-ccaat tcccc tacaa acttCG-3′, 5′-ccaat tcccc tacaa acttc atcct ccaaa atCG-3′ and 5′-ccaat tcccc tacaa acttc atcct ccaaa atcac cCG-3′). The forward primer and three reverse primers were used in the same reaction. The touchdown PCR was initiated at an annealing temperature of 62°C and decreased by 1°C per cycle for 10 cycles followed by 30 additional cycles at an annealing temperature of 52°C. 83 bp-MSP (primers for the 83 bp amplicon of methylated-*p16*, 5′-cgatt ttagg ggtgt tatat tcgtt aagtg ttc-3′ and 5′-aaaca aaaaa acgcc gtaaa cgaat actcg-3′; primers for the 88 bp amplicon of unmethylated-*p16*, 5′-gtgat tttag gggtg ttata tttgt taagt gtttg-3′ and 5′-ttcca aacaa aaaaa cacca taaac aaata ctca-3′). The following PCR conditions were used: 95°C for 15 min, and 38 cycles of 95°C for 30 s, 59°C for 30 s, and 72°C for 30 s followed by 72°C for 10 min. The 88 bp-MSP assay was used to detect unmethylated *p16* alleles in all tested tissue samples.

### Quantitative RT-PCR for detection of *p16* mRNA

To detect the relative *p16* mRNA level in gastric biopsy, Power SYBR Green PCR Master Mix (ABI) was used in the quantitative RT-PCR as described previously [13].

### Detection of *p16* methylation with 392 bp DHPLC assay

The fully methylated-*p16* alleles in gastric tissues was detected using the 392 bp DHPLC assay and fluorescence detector as we reported previously [29].

## Results

### Nucleosome positioning across p16 CpG island in human gastric carcinoma (GC) cell lines

To validate prediction data on the *p16* nucleosome occupancy in the human genome (Figure 1A) [28], we characterized nucleosome position within *p16* CpG island in the *p16*-methylated human GC cell line AGS and the *p16*-unmethylated cell line MGC803 (detected by 392 bp DHPLC) using the mono-nucleosomal DNA as the template. The chromatin accessibility was measured by a set of quantitative PCR to verify the predicted nucleosome positioning (Figure 1B). We found that amplicons of primer-2/4/7/8/9 sets were highly resistant to *MNase* digestion in the AGS cell line suggesting the presence of a nucleosome core in these regions. In contrast, amplicons of primer-1/3/5/6 sets showed *MNase* digestion hypersensitive suggesting the existence of nucleosome-free region such as linker DNA (Figure 1C). These patterns are consistent with the predicted nucleosome occupancy well [15], [30]. Interestingly, the nucleosome signal in the amplicons of primer-2, -8, -9 in the *p16*-methylated AGS cells was significantly stronger than that in the *p16*-umethylated MGC803 cells. In addition, the hypersensitive region of the primer set-5, which centered in the vicinity of the TSS of *p16*, showed virtually no signals after PCR and might indicate the preferred position of the linker DNA in the *p16*-methylated AGS cells. On the contrary, the hypersensitive region around the TSS was not detectable in the *p16*-unmethylated MGC803 cells, which suggests the existence of a transcription factor complex.

### Nucleosomes correlated with extension pattern of *de novo* methylation of *p16* CpG islands in human gastric carcinogenesis

**Figure 2:**
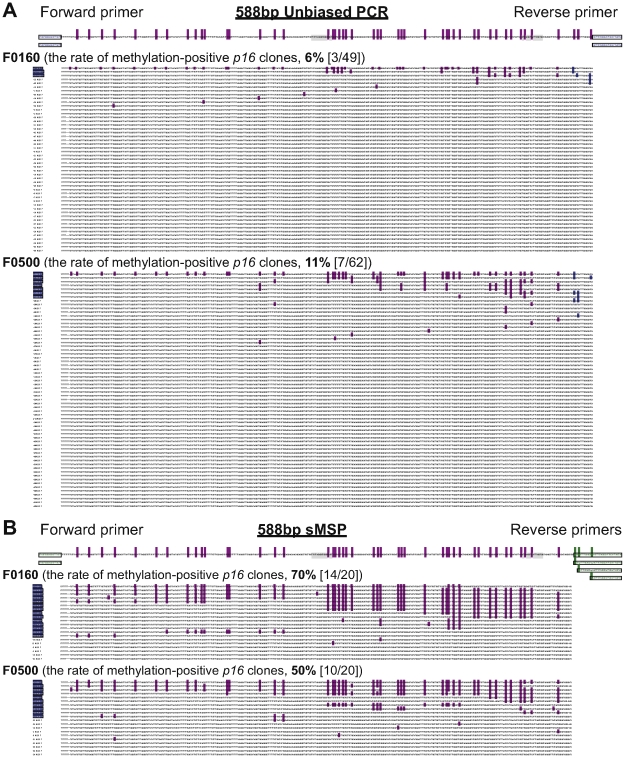
Results of bisulfite-sequencing of the 588 bp fragment of the *p16* CpG islands. (**A**) Sequences of the methylation-positive and negative clones from the 588 bp unbiased PCR products amplified using a CpG-free primer set and bisulfite-treated genomic DNA templates of two representative gastric carcinoma tissues containing methylated *p16* by the 115 bp MethyLight assay [11]; (**B**) Sequences of the methylation-positive and negative clones from the 588 bp sMSP products. Purple bars, methylated CpG sites; Blue bars, seeding methylated CpG sites detected in the unbiased PCR products; Green bars, seeding methylated CpG sites used to design the sMSP primers. Each row presents one clone; Clone markers of methylation-positive *p16* molecules (containing more than three methylated-CpG sites) are highlighted with blue color. The standard sequence of the fully methylated *p16* amplicon and the primer-matched regions in each assay are demonstrated on the top part.

To illustrate the progressive *p16* methylation pattern during tumorigenesis and its correlation with nucleosome, we first used the CpG-free primer set to amplify both the methylated and unmethylated 588 bp fragments ranged from the proximal promoter nucleosome to the coding nucleosome around the *p16* TSS after bisulfite treatment. Bisulfite-sequencing showed that the 3 of 49 (6%) and 7/62 (11%) clones were methylation-positive clones (containing more than three methylated CpG sites) in two representative GC samples F0160 and F0500 containing methylated-*p16*, respectively (Figure 2A). Interestingly, the methylated CpG sites in the coding nucleosome were observed among all methylation-positive *p16* clones. Unfortunately, the ratio of methylation-positive clones was too low to get enough number of methylation-positive clones using bisulfite-sequencing to make a solid *p16* methylation pattern in these samples because of the existence of large proportion of background methylation-negative *p16* alleles.

To better clarify how aberrant methylation in *p16* promoter is initially established from only a small portion of cells undergoing *de novo* methylation, we sought to develop a more robust assay capable of enriching initially methylated *p16* molecules in gastric biopsy samples with high sensitivity. It is reported that three 3′-CpG sites in the *p16* intron-1 are the methylation seeding sites in mammary epithelial cell strain (Supplementary Figure S1A, under-blue-lined sites) [14]. In addition, methylation at these seeding sites is frequently observed in all fully methylated *p16* clones and most partially methylated clones from the primary GC tissues in our previously published study (Figure S1B, blue-dots) [29]. This phenomenon was observed again in the unbiased PCR sequencing in the present study (Figure 2A, blue-dots). Thus, we designed a novel seeding methylation-specific PCR (sMSP) to enrich *p16* molecules (588 bp) containing at least one of the seeding methylated-CpG sites (Figure 2B, green-bars). A CpG-free forward primer and three reverse primers matched to one of three seeding methylated-CpG sites were used in the sMSP assay. To further support and confirm the methylation data obtained using sMSP, we sequenced the sMSP products and compared the results with those obtained from the unbiased 588 bp products. As shown in Figure 2B, the positive rate of methylation-positive *p16* clones increased from 6% (3/49) to 70% (14/20) and 11% (7/62) to 50% (10/20) for the sample F0160 and F0500, respectively (Figure 2B). Most importantly, the *p16* methylation pattern obtained using the sMSP-sequencing was similar to that using the unbiased PCR-sequencing. Thus, the sMSP assay was used to analyze dynamic processes of initiation and extension of *p16* methylation during gastric carcinogenesis as described below.

**Figure 3:**
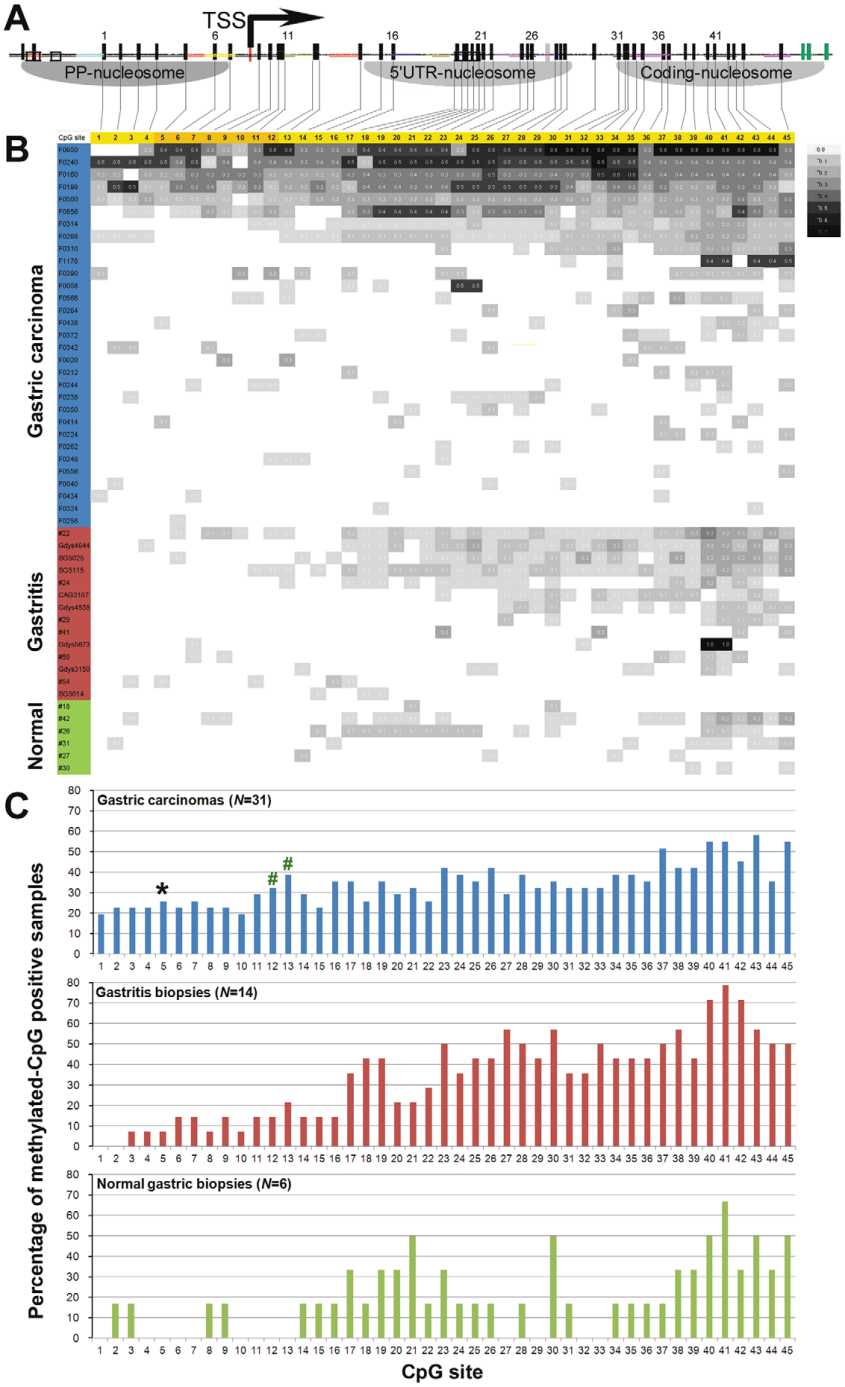
Relationship between nucleosome positioning and methylation of each CpG site within *p16* CpG island in human gastric mucosa samples (*N* = 51). (**A**) The detected nucleosome occupancy within a *p16* CpG island as displayed in Figure 1B; three seeding methylation CpG sites at 46, 47, and 48 locations are marked with purple color; (**B**) A gray-graded representation of the average methylation density at individual CpG sites within the *p16* promoter and exon-1 region based on the results of all informative clones obtained from each sample using the sMSP-sequencing assay; The methylation density of each CpG site in each tested sample was labeled, 0.1∼0.7 mean 10%∼70%; (**C**) The positive rate of methylated-CpG at each CpG site in the sequenced gastric tissue samples with various pathological changes. *, The positive rate at the site-5 in GCs is statistically significantly higher than in gastritis and normal samples (Fish exact test, *P*<0.02). #, The positive rate at the site-12 and 13 GCs is significantly higher than in gastritis and normal when the proportion value of each sample was used in the Mann Whitney test (*P* = 0.043, two-sides).

Using the sMSP assay, we first analyzed the *p16* methylation in 99 gastric tissue samples with various pathological changes. Results showed that 98 of 99 samples were *p16* methylation informative and that the sMSP-positive rate in GC samples (36/40) was significantly higher than that of normal gastric biopsies (7/13) or that of chronic gastritis lesions (19/45) (90% vs. 54% or 42%, *P* = 0.005 or 0.000). Then, all these sMSP products were extensively clone-sequenced (approximately 20 clones per sample). Sequencing information was obtained from 31 GC, 14 gastritis, and 6 normal samples (Figure 3). The average proportion of methylation of individual CpG site within the tested *p16* fragment was calculated based on all informative clones for each sample and displayed in the corresponding gray-graded pattern (from dark to blank, 8 grades; Figure 3B). The noise-level of sporadic methylated CpG sites was randomly observed among 45 analyzed CpG sites (except three anchoring methylated CpG sites at the reverse primers) in normal gastric tissues, gastritis lesions, and GCs. To standardize the primary data on methylation prevalence in different groups of gastric tissues, we defined a CpG site in a sample is methylated, when methylation of the CpG site was observed in one of all informative clones from the sample and used it to calculate the methylation positive rate of each CpG site among GC, gastritis, and normal samples (Figure 3C). The methylation positive rate in the promoter region in GCs is higher than in gastritis and normal samples (Fish exact test, GC [8/31] versus gastritis/normal [1/20] for the site-5, *P*<0.02; GC [6/31] versus gastritis/normal [0/20] for the site-1, *P* = 0.07). When the density of methylation was calculated in statistical analysis, the proportion of methylated CpG at the site-12 and site-13 in GCs was also significantly higher than in normal and gastritis samples (Mann and Whitney test, *P* = 0.043, two-sides). A gradual extension trend of *de novo* methylation within the CpG islands from the exon-1 coding-nucleosome to the proximal promoter (PP)-nucleosome was also observed from gastritis lesions to GCs.

To analyze the relationship between *p16*-methylation and its transcription in gastric tissues, we quantified the relative *p16* mRNA level in these tissues. Methylation status of *p16* CpG islands in these samples was also detected using the 392 bp-DHPLC assay that was established to detect methylation in 35 CpG sites, including three seeding methylation sites [29]. The relative *p16* mRNA level in 14 GCs with methylated-*p16* was significantly lower than that in 26 GCs without methylated-*p16* [ΔCt (*mean* ± *SD*), 12.79±2.77 versus 8.51±3.32, *P*<0.001; higher ΔCt value means lower mRNA level]. The methylated-*p16* was only detected in one of 29 gastritis/normal samples in which *p16* mRNA was not detected. The positive detection rate of *p16* mRNA in GC samples without *p16* methylation was significantly higher than that in gastritis/normal samples without *p16* methylation (26/26 versus 15/28, *P*<0.001). The average *p16* mRNA level [ΔCt, 13.37±3.28] in these 15 *p16*-expressed gastritis/normal samples was also much lower than that in the GC samples with or without *p16* methylation.

### *p16* Full methylation extended to the proximal promoter might be a tumor-specific event

**Figure 4:**
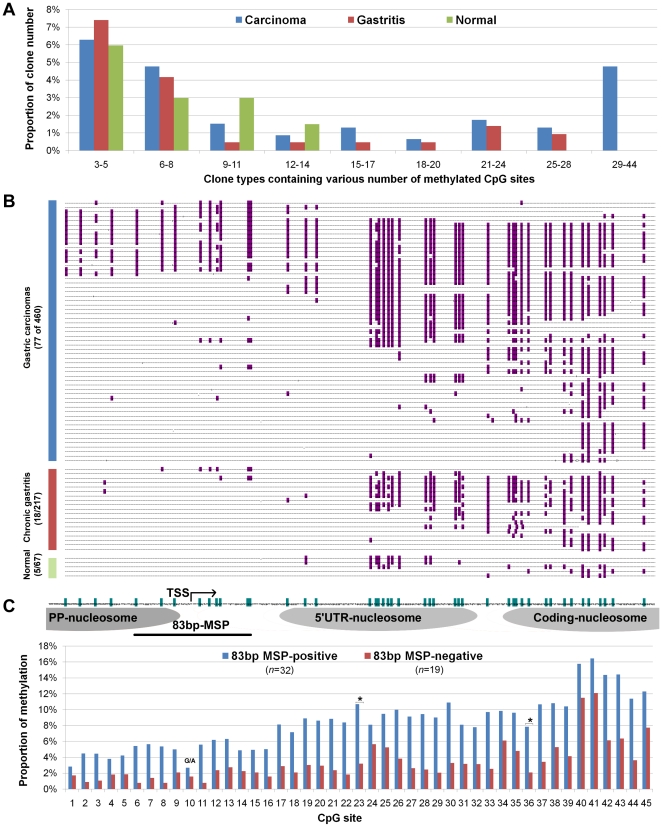
Comparison of methylation status of *p16* CpG island in gastric carcinomas (GC), chronic gastritis, and normal gastric mucosa using sMSP-sequencing. (**A**) Proportion of clone groups containing different number of methylated-CpG sites within the bisulfite-sequenced clones; (**B**) The proportion of clones containing ≥6 methylated-CpGs in sMSP clones from GCs (16.7%) was significantly higher than that from gastritis (8.3%) or normal biopsies (7.5%) (77/460 vs. 18/217 or 5/67, *p* = 0.003 or 0.050). The fully methylated-CpG cluster in the proximal promoter (PP)-nucleosomal region was observed only in GC samples. Most *p16* molecules with promoter methylation are fully methylated. (**C**) The average methylation frequency of each CpG site within the 45 tested CpGs in the 83 bp-MSP-positive samples (*n* = 32) and the negative samples (*n* = 19). The G in the CpG site-10 is a G/A polymorphism.

To clarify if the exact *p16* promoter methylation is tumor-specific, we further analyzed the methylation-positive clones (containing ≥3 methylated-CpG sites) obtained from gastric tissues using sMSP-sequencing. Our study showed that the largest number of methylated-CpG sites within a single clone was never higher than 14 among 67 clones from 6 sMSP-sequenced normal gastric samples, and the number of methylation sites was never more than 28 among 217 clones from 14 gastritis samples. In contrast, clones containing more than 29 methylated-CpGs were found among 460 clones from 31 GCs (Figure 4A). The proportion of clones with ≥6 methylated-CpG sites from GCs (16.7%) was also significantly higher than that from gastritis (8.3%) or normal biopsies (7.5%) (77/460 vs. 18/217 or 5/67, *p* = 0.003 or 0.050) (Figure 4B). The methylated-CpG cluster in the PP-nucleosomal region was observed only in GC samples. Moreover, we found that most *p16* molecules containing promoter CpG methylation were fully methylated across the CpG island. Therefore, we reasoned that the methylation within this region might be a tumor-specific event.

To exclude if the sMSP assay itself might lead to the promoter-specific bias, we developed an 83 bp-MSP assay capable of detecting CpG methylation across the linker region between the PP- and 5′UTR-nucleosomes (Figure 4C). Results of the 83 bp-MSP assay confirmed the validity of the sMSP analysis. The methylated-*p16* by 83 bp-MSP was detected in 33 of 55 sMSP-positive samples, but only in 1 of 6 sMSP-negative samples (*P*<0.04) (Figure S2). The average methylation frequency at all 45 tested CpGs in the 83 bp-MSP-positive samples (*N* = 32) is higher than that observed in the negative samples (*N* = 19) (Figure 4C). Then, we used the 83 bp-MSP assay to detect *p16* methylation in more GC samples and gastritis/normal biopsy samples, and found that the methylated-*p16* was detected in 60.3% (41/68) GCs, 42.1% (24/57) gastritis/normal gastric tissues, respectively (*p* = 0.043).

### *p16* Methylation positively correlated with the existence of *H. pylori* in normal/gastritis

**Figure 5:**
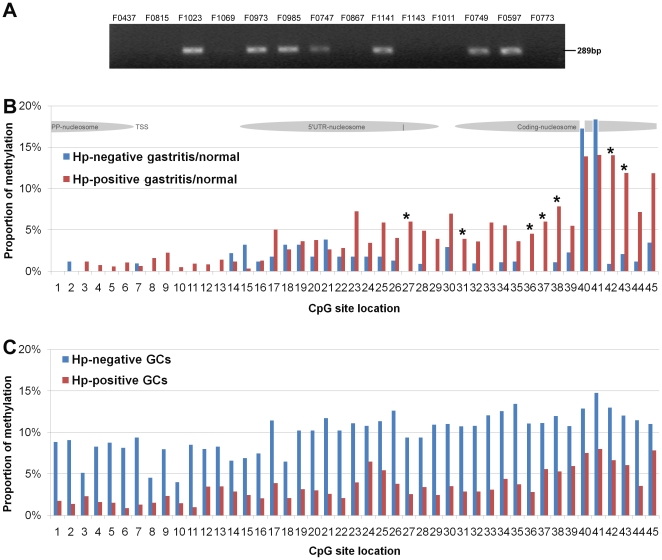
Correlation between *H. pylori* infection and the average proportion of methylated-CpG site at each CpG site within *p16* CpG island. (**A**) Result of 289 bp PCR products of *H. pylori*-specific *23S rDNA* from 14 representative gastric samples detected as previous report [22]; (**B**) The methylation proportion of each *p16* CpG site in the *H. pylori*-positive normal/gastritis biopsies (*N* = 13) and the *H. pylori*-negative samples (*N* = 7); The gray ovals represent the nucleosome position; (**C**) The methylation proportion of each *p16* CpG site in the *H. pylori*-positive GC samples (*N* = 17) and the *H. pylori*-negative GC samples (*N* = 9).

*H. pylori* infection is the main cause of gastritis; *p16* methylation in this lesion is *H. pylori* density- dependent [21]. In the present study, we directly detected *H. pylori* genomic DNA in these analyzed gastric samples using a *H. pylori*-specific *23S rDNA* PCR assay (Figure 5A), and found 46 samples containing *H. pylori* DNA among 64 sequenced samples (17 of 26 GCs and 13 of 20 gastritis/normal). The sMSP-positive rate of *p16* methylation in the *H. pylori*-positive samples was much higher than *H. pylori*-negative samples (30/34 vs. 16/30, *P*<0.001).

We further analyzed the relationship between the existence of *H. pylori*-DNA and the methylation status of 45 CpG sites in *p16* CpG island among the 46 sMSP-positive samples. As expected, we found that the methylation density of CpG site-27/31/36/37/42/43 within the sequenced *p16* fragment in the *H. pylori*-positive normal/gastritis samples (*N* = 13) was statistically significantly higher than that in the *H. pylori*-negative ones (*N* = 7), especially in the exon-1 coding-nucleosomal region (Wann Whitney test, *P*≤0.040; Table 1 and Figure 5B). Unexpectedly, we found that such difference could not be observed in GC samples. In contrast, the density of methylated-CpG sites in the *H. pylori*-negative GC samples (*N* = 9) was even higher than those observed in the *H. pylori*-positive samples (*N* = 17), especially across the PP- and 5′UTR-nucleosomal regions (Figure 5C).

**Table 1:** Comparison of the proportion of methylation at individual CpG site in the *p16* CpG islands in GCs and gastritis/normal biopsies containing or not containing *H. pylori* (*Hp*)-specific DNA.

	Normal and Gastritis (%)				Gastric carcinomas (%)			
	Average		Median (25th–75th)		Average		Median (25th–75th)	
	*Hp* (−) (*N* = 7)	*Hp* (+) (*N* = 13)	*Hp* (−) (*N* = 7)	*Hp* (+) (*N* = 13)	*Hp* (−) (*N* = 9)	*Hp* (+) (*N* = 17)	*Hp* (−) (*N* = 9)	*Hp* (+) (*N* = 17)
CpG site-1	0	0	0 (0–0)	0 (0–0)	8.9	1.8	0 (0–0)	0 (0–5.0)
CpG site-2	1.2	0	0 (0–0)	0 (0–0)	9.1	1.4	0 (0–7.1)	0 (0–0)
CpG site-3	0	1.2	0 (0–0)	0 (0–0)	5.1	2.3	0 (0–0)	0 (0–0)
CpG site-4	0	0.8	0 (0–0)	0 (0–0)	8.3	1.6	0 (0–0)	0 (0–0)
CpG site-5	0	0.6	0 (0–0)	0 (0–0)	8.8	1.5	0 (0–9.1)	0 (0–0)
CpG site-6	0	1.1	0 (0–0)	0 (0–0)	8.1	0.9	0 (0–6.3)	0 (0–0)
CpG site-7	1.0	0.6	0 (0–0)	0 (0–0)	9.4	1.3	0 (0–5.0)	0 (0–0)
CpG site-8	0	1.6	0 (0–0)	0 (0–0)	4.6	1.5	0 (0–0)	0 (0–0)
CpG site-9	0	2.3	0 (0–0)	0 (0–0)	8.0	2.4	0 (0–0)	0 (0–0)
CpG site-10	0	0.5	0 (0–0)	0 (0–0)	4.0	1.5	0 (0–0)	0 (0–0)
CpG site-11	0	0.9	0 (0–0)	0 (0–0)	8.5	1.0	0 (0–5.0)	0 (0–0)
CpG site-12	0	0.8	0 (0–0)	0 (0–0)	8.0	3.5	0 (0–5.0)	0 (0–0)
CpG site-13	0	1.4	0 (0–0)	0 (0–0)	8.3	3.5	0 (0–5.0)	0 (0–5.3)
CpG site-14	2.2	1.2	0 (0–0)	0 (0–0)	6.6	2.9	0 (0–0)	0 (0–6.7)
CpG site-15	3.2	0.3	0 (0–5.0)	0 (0–0)	6.9	2.5	0 (0–0)	0 (0–0)
CpG site-16	1.2	1.3	0 (0–0)	0 (0–0)	7.5	2.1	0 (0–5.0)	0 (0–0)
CpG site-17	1.8	5.0	0 (0–0)	0 (0–7.7)	11.4	3.9	0 (0–10.8)	0 (0–7.1)
CpG site-18	3.2	2.7	0 (0–5.0)	0 (0–6.7)	6.5	2.1	0 (0–5.0)	0 (0–0)
CpG site-19	3.2	3.6	0 (0–5.0)	0 (0–7.1)	10.2	3.2	0 (0–5.0)	0 (0–5.3)
CpG site-20	1.8	3.8	0 (0–0)	0 (0–7.7)	10.2	3.0	0 (0–5.0)	0 (0–0)
CpG site-21	3.8	2.7	0 (0–6.3)	0 (0–6.7)	11.7	2.6	2.7 (0–5.9)	0 (0–0)
CpG site-22	1.8	2.8	0 (0–0)	0 (0–6.7)	10.2	2.1	0 (0–5.0)	0 (0–0)
CpG site-23	1.8	7.2	0 (0–0)	7.1 (0–13.0)	11.1	4.0	0 (0–10.0)	0 (0–7.1)
CpG site-24	1.8	3.4	0 (0–0)	0 (0–7.1)	10.8	6.5	0 (0–10.0)	0 (0–5.9)
CpG site-25	1.8	5.9	0 (0–0)	0 (0–8.7)	11.4	5.4	0 (0–15.0)	0 (0–5.3)
CpG site-26	1.3	4.0	0 (0–0)	0 (0–7.1)	12.6	3.8	0 (0–7.1)	0 (0–5.3)
CpG site-27	0	6.0	0 (0–0)	5.9 (0–7.1)^a^	9.4	2.6	0 (0–5.0)	0 (0–0)
CpG site-28	0.9	4.9	0 (0–0)	5.9 (0–7.1)	9.4	3.4	0 (0–5.0)	0 (0–6.3)
CpG site-29	0	3.9	0 (0–0)	0 (0–6.7)	11.0	2.5	0 (0–10.0)	0 (0–0)
CpG site-30	2.9	7.0	0 (0–3.1)	7.1 (0–11.8)	11.0	3.5	0 (0–15.0)	0 (0–6.7)
CpG site-31	0	3.9	0 (0–0)	0 (0–7.1)^b^	10.7	2.9	0 (0–10.0)	0 (0–0)
CpG site-32	1.0	3.6	0 (0–0)	0 (0–6.7)	10.8	2.9	0 (0–15.0)	0 (0–0)
CpG site-33	0	5.9	0 (0–0)	4.3 (0–7.1)	12.1	3.1	0 (0–10.0)	0 (0–0)
CpG site-34	1.1	5.6	0 (0–0)	0 (0–6.7)	12.6	4.4	0 (0–10.0)	0 (0–0)
CpG site-35	1.2	3.6	0 (0–0)	0 (0–6.7)	13.4	3.7	0 (0–15.0)	0 (0–0)
CpG site-36	0	4.6	0 (0–0)	0.5 (0–7.7)^c^	11.1	2.8	0 (0–15.0)	0 (0–0)
CpG site-37	0	6.0	0 (0–0)	7.1 (0–10.0)^d^	11.1	5.6	5.0 (0–10.0)	0 (0–11.1)
CpG site-38	1.1	7.9	0 (0–0)	8.3 (0–11.8)	12.0	5.3	0 (0–16.2)	0 (0–11.1)
CpG site-39	2.3	5.5	0 (0–3.8)	0 (0–8.7)	10.8	6.0	0 (0–15.0)	0 (0–12.5)
CpG site-40	17.3	13.9	0 (0–10.4)	16.7 (6.7–21.4)	12.9	7.5	8.1 (0–30.0)	0 (0–11.1)
CpG site-41	18.4	14.1	7.7 (0–10.4)	13.3 (7.1–21.7)	14.8	8.0	8.1 (0–30.0)	0 (0–12.5)
CpG site-42	0.9	14.1	0 (0–0)	15.4 (13.3–17.4)^e^	13.0	6.6	0 (0–30.0)	0 (0–12.5)
CpG site-43	2.1	11.9	0 (0–3.1)	13.0 (0–20.0)^f^	12.0	6.1	2.7 (0–20.0)	0 (0–6.7)
CpG site-44	1.2	7.2	0 (0–0)	6.7 (0–10.4)	11.5	3.6	2.7 (0–15.0)	0 (0–0)
CpG site-45	3.5	11.9	0 (0–3.8)	13.3 (0–23.1)	11.0	7.8	5.4 (0–15.0)	0 (0–12.5)

a, b, c, d, e, f Wann Whitney test, *P* = 0.012, 0.040, 0.023, 0.010, 0.018, 0.003, 0.034.

## Discussion

Over the past decade, numerous evidences indicate that DNA methylation, nucleosome positioning, and histone modification form a complex regulatory network to modulate gene expression and genome function epigenetically [31], [32]. The interplay between DNA methylation and nucleosome occupancy has been addressed recently [1], [25], [26]. It is reasonable to speculate that the nucleosome, which typically consists of a 147 bp DNA fragment and an octamer of histones H2A, H2B, H3, and H4, may act as the basic *de novo* methylation unit in the initiation and extension stages of methylation [26]. It was determined that *RASSF1A* hypermethylation spreads progressively from its exon-1 to the promoter during breast tumorigenesis [33]. In the present study, the natural dynamics of this interplay were analyzed in a number of human gastric tissues in different stages of carcinogenesis. We found that *de novo* methylated-CpGs within *p16* CpG islands were concentrated and extended in a nucleosome-specific pattern. This pattern of *de novo* methylation (extension) of *p16* CpG islands observed in human gastric mucosa was related to the development of gastric carcinomas. To the best of our knowledge, this is the first report to address the gradual progression and accumulation of *de novo* methylation of *p16* CpG islands *in vivo*.

It is generally considered that the aberrant full methylation of CpG islands of the tumor suppressor genes of cultured cell lines and cancer cells has been established, and that fraction of cells that undergo *de novo* methylation in cellular heterogeneous tissues is limited. However, the exact status of these epigenetic events in tissues in different pathological stages of carcinogenesis is not well studied. The major obstacle in characterizing aberrant *de novo* methylation patterns of CpG islands *in vivo* is the difficulty associated with capturing DNA molecules in the initiation and extension stages of methylation. As we display in Figure 2A, the efficiency of the unbiased bisulfite-sequencing is very low. A methylation enrichment technology should be used to determine the initiation and extension pattern of *p16* methylation. Although 5-methylcytosine antibody or methylated-DNA binding protein immunoprecipitation (MeDP) is the regular approach for enrichment of methylated-DNA, however, whether this approach is suitable to enrich DNA fragments with methylation initiation in small tissue samples, such as gastric mucosa biopsies, is unknown. Thus, we have to establish other sensitive method to enrich the methylation-positive *p16* molecules. It has been reported that three CpG sites in the *p16* intron-1 may be the seeding methylation sites for the initiation of *p16* methylation [14]. Our present and previously reported studies using bisulfite sequencing of 588 bp and 392 bp unbiased PCR products show that these CpG sites are indeed initial methylation sites, as we demonstrate in Figure 2A (blue bars) and Supplementary Figure S1 (blue dots), because methylation of the seeding sites can be observed not only in the fully or extensively methylated clones, but also in most of these clones containing a few methylated CpG sites. Therefore, based on these observations, we developed a novel approach, sMSP, to enrich the *p16* molecules that are methylated at least at one of three seeding methylation sites in tissue samples. In two representative GC samples, we found the *p16* methylation patterns obtained using the unbiased bisulfite-sequencing and sMSP were similar, but the efficiency of sMSP to capture the methylation-positive molecules is about 5 times of the unbiased assay.

Extensive clone sequencing of the sMSP products demonstrated clearly that in normal gastric tissue the majority of the samples did not show CpG methylation or contained sporadically methylated-CpG sites in the exon-1 coding-nucleosome region. In contrast, the gastritis and GC samples showed various degrees of methylation in the same region. Extensive methylation in both the promoter and the exon-1 regions was observed in the majority of the GC specimens. We propose that a methylation wave progressively extends from the *p16* exon-1 coding-nucleosome to its promoter nucleosome *in vivo*. The overall *p16* methylation profiles constructed from a panel of clinical specimens may approximate the natural extension pattern of *de novo* methylation of *p16* CpG islands in gastric carcinogenesis.

Other studies have revealed that the methylation process in the CpG islands of genes in cultured cells contain ‘hot spots’ that are considerably more prone to methylation [16]. Our *in vivo* study identified similar ‘hot spots’ in similar locations observed in clinical specimens. We also propose the possibility that the focal ‘hot spots’ and the wave-extension pattern could directly reflect the nucleosome positioning. This compelling observation correlates well with recent findings of increased methylation density within a nucleosome of a susceptible CpG island [1]. The underlying molecular mechanism could be linked to DNMT preferentially targeted nucleosome-bound DNA [1], [34]. Histone modifications, such as H3K27 trimethylation, maybe necessary for establishment of *p16* methylation [35]. The dependency of *de novo* methylation of CpG islands on the presence of nucleosomes should be studied further.

It is suggested that *p16* methylation precedes the formation of pre-neoplastic lesions, which indicate that epigenetic events might play a role in the induction of oncogenic pathways in early stages of carcinogenesis [6], [7]. In fact, *p16* methylation has the potential to be the risk prediction biomarker for cancers in the stomach and other organs [8]–[10]. However, extensive aberrant *p16* methylation in the exon-1 region was also observed in gastric tissues with chronic gastritis as determined using the classic 150 bp-MSP assay [21]. Unlike the stable *p16* methylation in cancer cells [19], *p16* methylation in gastritis lesions is unstable and *H. pylori*-dependent as indicated through the 150 bp-MSP assay [20], [21]. Various CpG islands, as well as different regions within the same CpG island, show different degrees of susceptibility to methylation [36]. To develop an assay capable of using *p16* as a tumor biomarker, it would be necessary to search for distinct methylation regions within a *p16* CpG island where methylation is stable and cancer-specific. In the present study, we found that methylation of the *p16* proximal promoter nucleosome was only observed in GC samples making this location a possible candidate for further development of a tumor biomarker, and that nearly all of the *p16* molecules with promoter methylation were found to be fully methylated in GC samples. These phenomenon suggest that fully methylated-*p16* molecules (from the exon-1 to the promoter) maybe not only more stable than partially methylated ones, but also tumor-specific. This might account for the wide range of stability observed in various methylated-*p16* alleles from gastric tissues with different lesions.

The inversed relationship between *p16* methylation and its transcription repression was firmly established in cultured cell lines [3], [5], [13]. Because most GC samples were sMSP-positive (36/40), we had to use other assay to detect *p16* methylation in GC samples to analyze the methylation-repression relationship in GC samples. The 392 bp DHPLC assay was established to detection methylation status of 35 CpG sites in the *p16* exon-1, including three seeding methylation CpG sites, as demonstrated on the supplementary Figure S1B [13]. Thus we used the 392 bp-DHPLC assay was selected to detect *p16* methylation in the present study. The significantly inversed relationship was observed between GC samples with and without *p16* methylation. In addition, *p16* is one of weakly transcribed tumor suppressor genes in normal tissues including the stomach. Both upregulation of *p16* in some cell population and methylation-silence in other cell population can often be observed in cervical cancers and precancerous tissues [10]. In this study, we also observed both higher *p16* mRNA level and higher sMSP-positive rate in GC samples than in gastritis/normal samples.

It is well recognized that *H. pylori* infection can induce gastritis, gastric ulcers, and gastric MALT lymphoma. Although *H. pylori* infection increases GC risk, the causal relationship between GC and *H. pylori* infection has not yet been established. We previously evaluated the density of *H. pylori*-like microorganisms in gastric mucosa containing gastritis or dysplasia lesions under microscope and found that the density positively correlated with the prevalence of *p16* methylation [21]. In the present study, we directly detected the exact existence of *H. pylori*-specific *23S rDNA* in the analyzed DNA samples and found that the methylation at the *p16* exon-1 coding region did positively correlate with *H. pylori* infection in normal gastric biopsies and gastritis lesions. Surprisingly, such correlation could not be observed in GC specimens. In contrast, an inversed relationship was observed between *H. pylori* infection and methylation at all 45 tested CpG sites in GCs. This result implies that other causal factors may be the main contributors to the abnormal *p16* methylation seen in GC samples. It has been reported that polycyclic aromatic hydrocarbons (PAH) exposure may induce CpG site-specific methylation in the *p16* exon-1 region in peripheral blood lymphocytes from PAH exposed workers [37]. We have reported that *p16* methylation is a frequent epigenetic event observed when gastric carcinogenesis is induced in Wistar rats by the N-nitroso compound MNNG [7]. Similar carcinogenic N-nitroso compounds are known to be synthesized within the human stomach and have been reported to increase the risk of GC [38], [39]. Whether exposure of gastric chemical carcinogens induces the full methylation of *p16* in GC should be studied further.

In conclusion, we demonstrate that *p16* methylation evolves and progresses throughout the promoter in a stepwise “normal→gastritis→carcinoma" cascade, in which the methylation signature is directly connected to the position of nucleosome.

## Supporting Information

Table S1 ***p16*** **Primer sets of quantitative PCR for nucleosome positioning.**
(PDF)

Figure S1 **Results from bisulfite clone sequencing of the** ***p16*** **CpG islands.**
(PDF)

Figure S2 **Results of sMSP were confirmed with those of the 83 bp-MSP assay.**
(PDF)
