# Correction: Nucleosomes Correlate with *In Vivo* Progression Pattern of *De Novo* Methylation of *p16* CpG Islands in Human Gastric Carcinogenesis

**DOI:** 10.1371/annotation/eb15769c-aa54-4963-8a13-1dd7e8242319

**Published:** 2012-08-23

**Authors:** Zhe-Ming Lu, Jing Zhou, Xiuhong Wang, Zhenpo Guan, Hua Bai, Zhao-Jun Liu, Na Su, Kaifeng Pan, Jiafu Ji, Dajun Deng

Figure S2 is a duplicate of Figure S1. Please view the correct Figure 2S here: 

[^] 

