# Supplementary material for: Nucleosomes Correlate with In Vivo Progression Pattern of De Novo Methylation of p16 CpG Islands in Human Gastric Carcinogenesis
Source: PLoS One. 2012 Apr 25;7(4):e35928. doi: 10.1371/journal.pone.0035928 (PMC3338478; doi:10.1371/journal.pone.0035928)
Supplement: Table S1 — p16 Primer sets of quantitative PCR for nucleosome positioning. (PDF) [file pone.0035928.s001.pdf]

**Table S1**. *p16* Primer sets of quantitative PCR for nucleosome positioning

| | **Primer sequence (5'→3')** | **Amplicon size (bp)** |
|---|---|---|
| Primer-1 | F: taggg ggaca ctttc tagtc gtaca gg | 130 |
| | R: ggtgt ttggt gtcat aggga aa | |
| Primer-2 | F: tttcc ctatg acacc aaaca cc | 119 |
| | R: gcgtg tttga gtgcg ttcac tctg | |
| Primer-3 | F: agtga acgca ctcaa acacg c | 119 |
| | R: gttgg caagg aagga ggact | |
| Primer-4 | F: agtcct ccttc cttgc caac | 114 |
| | R: tccga gcact tagcg aatgt g | |
| Primer-5 | F: acttc agggg tgcca cattc | 124 |
| | R: accct gtccc tcaaa tcctct | |
| Primer-6 | F: tcccc ttgcc tggaa agata c | 121 |
| | R: ccacc ctctgg tgacc agc | |
| Primer-7 | F: caccg gaga agaa agagg a | 122 |
| | R: ccggc tccat gctgc tc | |
| Primer-8 | F: agagg gggag agcag gca | 127 |
| | R: cgccc gcacc tcctc tac | |
| Primer-9 | F: ggggt cgggt agagg aggt | 113 |
| | R: tgctc ccgct gcaga ccct | |

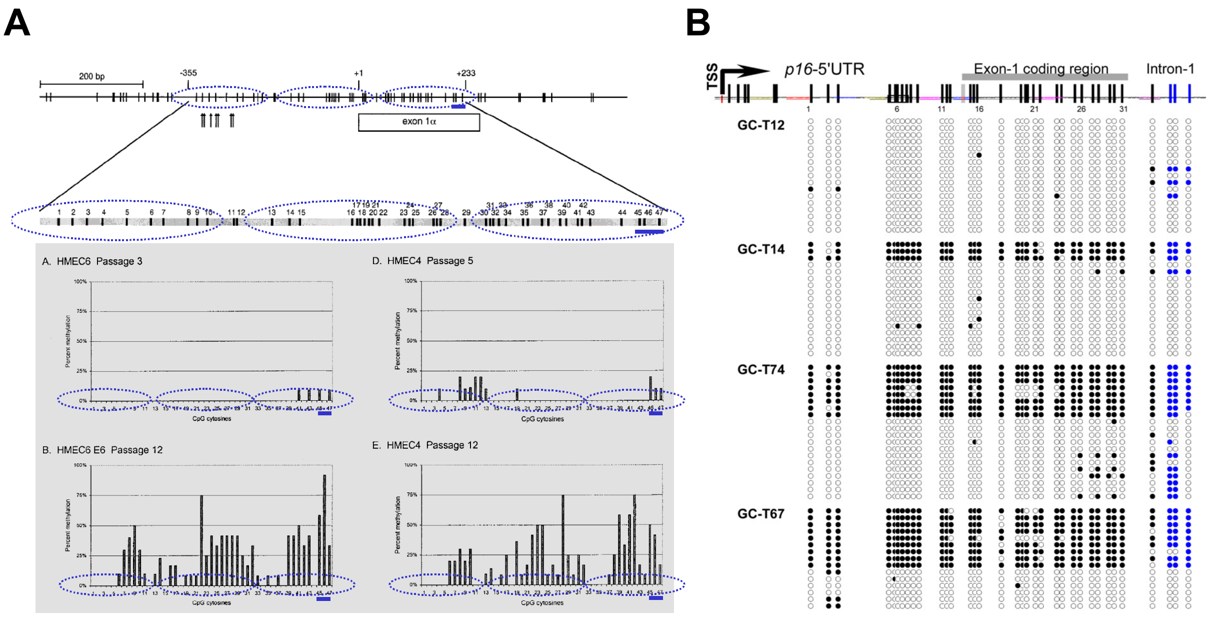


**Figure S1**. **Results from bisulfite clone sequencing of the *p16* CpG islands.** (**A**) The dynamic methylation status of individual CpG site in the whole *p16* CpG island during the immoralization of human mammary epithelial cell strains by stably HPV16 E6/E7 transfection as previously reported [14]. The information of nucleosome positioning (blue dash-line ovals) obtained in the present study was integrated into the published chart. Three seeding methylation sites were under-blue-lined. (**B**) The methylation status of the 392bp fragment of the *p16* CpG island in primary gastric carcinoma samples as previously reported [29]; Black dots (●) represent methylated-CpG sites open dots (○) represent unmethylated-CpG sites, blue dots (●) represent methylation at the three seeding methylation sites in intron-1. Each black bar represents a CpG site. TSS, transcription start site; the gray vertical bar, translation start site (atg).

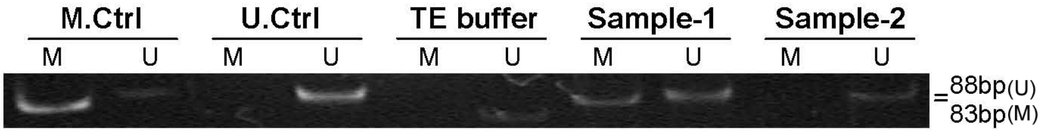


| | | 83bp MSP | |
|---|---|---|---|
| | | (+) | (-) |
| sMSP | (+) | **33** | **22** |
| | (-) | **1** | **5** |

($p$<0.04)

**Figure S2. Results of sMSP were confirmed with those of the 83bp-MSP assay**. The top panel shows the number of PCR products that exhibited methylated- and unmethylated-*p16* of two representative GC samples as determined using the 83bp-MSP assay. The lower panel compares the detection of methylated-*p16* in 61 gastric tissue samples using the 83bp-MSP and sMSP assays. The table indicated that the results of the 83bp-MSP correlated with those of sMSP significantly ($p<0.04$).
