# Supplementary material for: Nucleosomes Correlate with In Vivo Progression Pattern of De Novo Methylation of p16 CpG Islands in Human Gastric Carcinogenesis
Source: PLoS One. 2012 Apr 25;7(4):e35928. doi: 10.1371/journal.pone.0035928 (PMC3338478; doi:10.1371/journal.pone.0035928)
Supplement: Figure S1 — Results from bisulfite clone sequencing of the p16 CpG islands. (PDF) [file pone.0035928.s002.pdf]

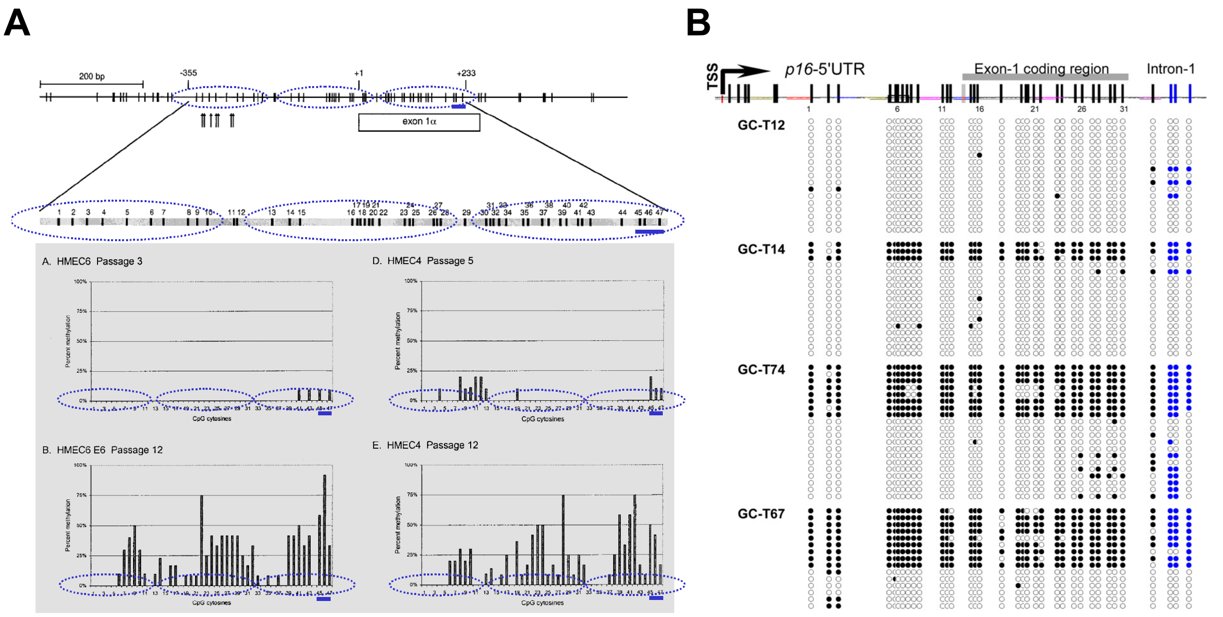

Figure S1. **Results from bisulfite clone sequencing of the *p16* CpG islands.** (**A**) The dynamic methylation status of individual CpG site in the whole *p16* CpG island during the immoralization of human mammary epithelial cell strains by stably HPV16 E6/E7 transfection as previously reported [14]. The information of nucleosome positioning (blue dash-line ovals) obtained in the present study was integrated into the published chart. Three seeding methylation sites were under-blue-lined. (**B**) The methylation status of the 392bp fragment of the *p16* CpG island in primary gastric carcinoma samples as previously reported [29]; Black dots (●) represent methylated-CpG sites open dots (○) represent unmethylated-CpG sites, blue dots (●) represent methylation at the three seeding methylation sites in intron-1. Each black bar represents a CpG site. TSS, transcription start site; the gray vertical bar, translation start site (atg).
