# Supplementary material for: Correction: Nucleosomes Correlate with In Vivo Progression Pattern of De Novo Methylation of p16 CpG Islands in Human Gastric Carcinogenesis
Source: PLoS One. 2012 Aug 23;7(8):10.1371/annotation/eb15769c-aa54-4963-8a13-1dd7e8242319. doi: 10.1371/annotation/eb15769c-aa54-4963-8a13-1dd7e8242319 (PMC3437787; doi:10.1371/annotation/eb15769c-aa54-4963-8a13-1dd7e8242319)
Supplement: Supplementary file 1 [file pone.eb15769c-aa54-4963-8a13-1dd7e8242319.s001.pdf]

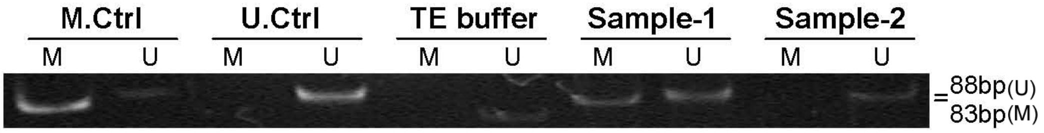

		83bp MSP	
		(+)	(-)
sMSP	(+)	33	22
	(-)	1	5

(p<0.04)

Figure S2. Results of sMSP were confirmed with those of the 83bp-MSP assay. The top panel shows the number of PCR products that exhibited methylated- and unmethylated-*p16* of two representative GC samples as determined using the 83bp-MSP assay. The lower panel compares the detection of methylated-*p16* in 61 gastric tissue samples using the 83bp-MSP and sMSP assays. The table indicated that the results of the 83bp-MSP correlated with those of sMSP significantly ($p<0.04$).
